# Mechanisms of Substrate Recognition by the Multispecific Protein Lysine Methyltransferase SETD6

**DOI:** 10.3390/life15101578

**Published:** 2025-10-10

**Authors:** Gizem T. Ulu, Sara Weirich, Jana Kehl, Thyagarajan T. Chandrasekaran, Franziska Dorscht, Dan Levy, Albert Jeltsch

**Affiliations:** 1 Institute of Biochemistry, University of Stuttgart, Allmandring 31, 70569 Stuttgart, Germany; 2 The Shraga Segal Department of Microbiology, Immunology and Genetics, Ben-Gurion University of the Negev, P.O.B. 653, Be’er-Sheva 84105, Israel; 3 The National Institute for Biotechnology in the Negev, Ben-Gurion University of the Negev, P.O.B. 653, Be’er-Sheva 84105, Israel

**Keywords:** SETD6, protein lysine methylation, protein lysine methyltransferase, enzyme specificity, enzyme–substrate interaction

## Abstract

The SETD6 protein lysine methyltransferase monomethylates specific lysine residues in a diverse set of substrates which contain the target lysine residue in a highly variable amino acid sequence context. To investigate the mechanism underlying this multispecificity, we analyzed SETD6 substrate recognition using AlphaFold 3 docking and peptide SPOT array methylation experiments. Structural modeling of the SETD6–E2F1 complex suggested that substrate binding alone is insufficient to restrict SETD6 activity to only one lysine residue, pointing to additional sequence readout at the target site. Methylation of mutational scanning peptide SPOT arrays derived from four different SETD6 substrates (E2F1 K117, H2A.Z K7, RELA K310, and H4 K12) revealed sequence preferences of SETD6 at positions −1, +2, and +3 relative to the target lysine. Notably, glycine or large aliphatic residues were favored at −1, isoleucine/valine at +2, and lysine at +3. These preferences, however, were sequence context dependent and variably exploited among different substrates, indicating conformational variability of the enzyme–substrate interface. Mutation of SETD6 residue L260, which forms a contact with the +2 site in the available SETD6-RELA structure, further demonstrated substrate-specific differences in recognition at the +2/+3 sites. Together, these findings reveal a versatile mode of peptide recognition in which the readout of each substrate position depends on the overall substrate peptide sequence. These findings can explain the multispecificity of SETD6 and similar mechanisms may underlie substrate selection in other protein methyltransferases.

## 1. Introduction

Post-translational modifications (PTMs) of proteins increase their chemical diversity, thereby overcoming the functional limitations of the twenty standard amino acids [1,2]. By altering protein structure and properties, PTMs regulate interactions of proteins with other biomolecules, including protein–protein and protein–DNA contacts. They can influence a wide range of properties, including protein localization, stability, turnover, conformation, and enzymatic activity, for example [1,2]. A large number of protein PTMs have been identified, including methylation, acetylation, phosphorylation, ubiquitination, glycosylation, and SUMOylation [1,2]. These covalent modifications are mediated by three main classes of regulatory proteins: writers, which catalyze the addition of a specific PTM to proteins; erasers, which remove these modifications; and readers, which recognize and bind to modified residues and propagate downstream signals. Together, these components form dynamic and reversible networks that control protein function and cellular signaling, playing critical roles in physiology and diseases.

Protein methylation involves transferring a methyl group from S-adenosyl-L-methionine (AdoMet) to specific amino acid residues, such as lysine, arginine, histidine, asparagine, and glutamine [2,3]. Methylation of lysine residues on histone and non-histone proteins is catalyzed by protein lysine methyltransferases (PKMTs) [3,4,5,6]. Most of them belong to the group of SET domain PKMTs [7], while others are 7β-strand enzymes [8]. SET Domain-Containing Protein 6 (SETD6) is a lysine mono-methyltransferase and a member of the SET domain PKMT family [9]. The SETD6 enzyme comprises an N-terminal domain, followed by the SET domain containing the active site, and a C-terminal domain. It was initially discovered to monomethylate K310 of RELA [10] but, in the following years, a large number of additional SETD6 substrates have been identified (Table 1). In a cellular context, SETD6 methylation events were shown to impact cell signaling, chromatin regulation, the binding affinity of modified transcription factors, and gene regulation [9]. Of note, SETD6 monomethylates its substrate proteins at defined lysine residues although they are located in very variable amino acid sequence contexts. In most cases, it methylates only one lysine residue on each substrate protein, indicating that the enzyme must interact in a specific manner with its distinct substrates, a feature that is best described as multispecificity.

It had been the aim of this work to understand the mechanism of the multispecific substrate recognition of SETD6. We started by modeling the interaction of SETD6 with the E2F1 protein by AlphaFold 3, showing that the binding of SETD6 and its substrates is likely not sufficient to restrict its access to only one lysine residue on the substrate protein. Next, we investigated the recognition of the sequence surrounding the target lysine by the active site cleft of SETD6 using peptide array methylation experiments. For this, we selected four different SETD6 substrates showing methylation, viz. E2F1 K117 [11] and H4 K12 [12], which were previously studied by us, as well as RELA K310 [10] and H2A.Z K7 [13], the first identified non-histone and histone substrates of SETD6. Taken together, these substrates nicely reflect the variability of sequences methylated by SETD6. Our data show that substrate peptides can interact with SETD6 in different modes, leading to the methylation of lysine residues in different sequence contexts and explaining the broad but still restricted substrate range of SETD6. Evidence with other protein methyltransferases suggests that this mechanism can be generalized to other enzymes of this class.

**Table 1:** Compilation of known SETD6 methylation substrates.

Target Protein	Target Residue	Sequence Context	Reference
RELA	K310	KRTYETFKSIMKKSP	[10]
H2A.Z	K7	-AGGKAGKDSGKAKT	[13]
WDR5	K207	TASGQCLKTLIDDDN	[14]
WDR5	K325	SAALENDKTIKLWKS	[14]
PLK1	K209	EYDGERKKTLCGTPN	[15]
PLK1	K413	IPIFWVSKWVDYSDK	[15]
H2A	K5	---SGRGKQGGKARA	[16]
H3	K14	ARKSTGGKAPRKQLA	[16]
H4	K12	KGGKGLGKGGAKRHR	[12,16]
PAK4	K473	SKEVPRRKSLVGTPY	[17]
BRD4	K99	LNLPDYYKIIKTPMD	[18]
MRPS23	K108	EKYTELQKLGETDEE	[19]
TWIST1	K33	RQQPPSGKRGGRKRR	[20]
E2F1	K117	GRGRHPGKGVKSPGE	[11]

## 2. Materials and Methods

### 2.1. Generation of the SETD6 L260A Mutant and SETD6 Purification

The mutant SETD6 (L260A) was generated in the plasmid encoding wild type (WT) His-SUMO tagged SETD6. Mutagenesis was performed using the megaprimer method [21]. The sequence of all plasmids was validated by Sanger sequencing (Supplementary Figure S1). For the protein overexpression, the plasmids encoding SETD6 WT and L260A were transformed into *E. coli* BL21-CodonPlus (DE3) cells (Novagen). Subsequently, the bacterial cells were cultivated in Luria–Bertani (LB) medium at 37 °C until an optical density (OD) at 600 nm of 0.6–0.8 was reached. The bacterial cultures were induced with 1 mM isopropyl β-D-1-thiogalactopyranoside (IPTG) (Biovectra) for 2.5 h at 20 °C. Afterwards, the cells were harvested by centrifugation at 4500 rpm. The cell pellet was dissolved in 1X STE buffer (10 mM Tris-HCl pH 8.0, 1 mM EDTA, and 100 mM NaCl) and centrifuged again.

For SETD6 purification, the cell pellet was resuspended in sonication buffer containing 30 mM sodium-phosphate buffer pH 7.2, 500 mM KCl, 0.2 mM DTT, 1 mM EDTA, 20 mM imidazole, and 10% glycerol and 1X protease inhibitor cocktail (PIC) including 1 mM AEBSF-HCl (Biosynth), 10 μM pepstatin (Roth), 0.4 μM aprotinin (Applichem), 15.1 μM E-64 (Applichem), 22.3 μM leupeptin (Alfa Aesar), and 50 μM bestatin (Alfa Aesar). The cells were harvested by 14 sonication steps using 4 cycles of 15 s each at 30% power (Bandelin Sonopuls). The samples were then centrifuged at 18,500 rpm for 90 min. The supernatant was passed through nickel–nitrilotriacetic acid–agarose beads (Genaxxon Bioscience), which had been equilibrated with sonication buffer. The protein-bound beads were then washed with 150 mL of sonication buffer. The bound proteins were eluted with sonication buffer containing 220 mM imidazole. The proteins underwent dialysis using a low-glycerol dialysis buffer I (20 mM HEPES pH 7.2, 200 mM KCl, 0.2 mM DTT, 1 mM EDTA, and 10% glycerol) for 2 h, after which they were transferred to a high-glycerol dialysis buffer II (containing 65% glycerol).

### 2.2. Synthesis of Peptide SPOT Arrays

Peptide arrays were prepared using the SPOT synthesis technique using an Autospot Multipep peptide array synthesizer (CEM Corporation, Matthews, Pittsburgh, PA, USA) [22]. Based on the Autospot Reference Handbook (CEM Corporation, Matthews, Pittsburgh, PA, USA) each peptide spot contains approximately 9 nmol of peptide in a diameter of 2 mm. The successful synthesis of peptide arrays was validated by Bromophenol blue staining [23,24].

### 2.3. Methylation of Peptide SPOT Arrays

The peptide arrays were pre-incubated for 5 min at room temperature on a shaker in methylation buffer (50 mM Tris-HCl pH 9 and 5 mM DTT). The SPOT arrays were then incubated with methylation buffer supplemented with 50 nM SETD6 and 0.5 mCi/mL [methyl-^3^H]-AdoMet (PerkinElmer LifeSciences) (82.3 Ci/mmol specific activity) for 1 h at 25 °C. Afterwards, the arrays were washed five times for 5 min with 100 mM NH_4_HCO_3_ buffer containing 1% SDS. After washing, the arrays were incubated in Amplify NAMP100V solution (GE Healthcare) for 5 min and then exposed to HyperfilmTM high-performance autoradiography films (GE Healthcare) in the dark at −80 °C for different exposure times. Finally, the films were developed using an Optimus TR developing machine.

### 2.4. Data Analysis

Each spot intensity was normalized based on the total intensity maximum and minimum of the corresponding array before calculating the average and the mean absolute error between replicates. For visualization, the discrimination factor for each amino acid at each position was calculated, which describes the preference of SETD6 for any specific amino acid residue at one site compared to all other residues at this place [23,24].

### 2.5. Protein Modeling

Modeling of SETD6–E2F1 complexes was performed using AlphaFold 3 (https://alphafoldserver.com/ accessed: 23 August 2025) [25] with a random seed. The full protein sequences of both proteins were used. The top models were downloaded and analyzed using Chimera 1.18 (https://www.rbvi.ucsf.edu/chimera/ accessed: 23 August 2025) [26]. The predicted local distance difference test (pLDDT) scores were directly extracted from the cif output file.

## 3. Results

SETD6 has been shown to methylate a wide range of proteins at lysine residues embedded in very different sequence contexts (Table 1), suggesting a low level of substrate recognition. On the other hand, it is not acting as a non-specific PKMT, as the labs working on SETD6 have found that numerous proteins are not targets of methylation, raising the interesting question of how the specific readout of different substrates is achieved. In this paper, we investigate the mechanistic foundations of the multispecific substrate recognition by SETD6, showing that it is based on variable substrate sequence-dependent conformations of the SETD6–peptide complex.

### 3.1. AlphaFold 3 Modeling of SETD6–E2F1 Complexes

**Figure 1:**
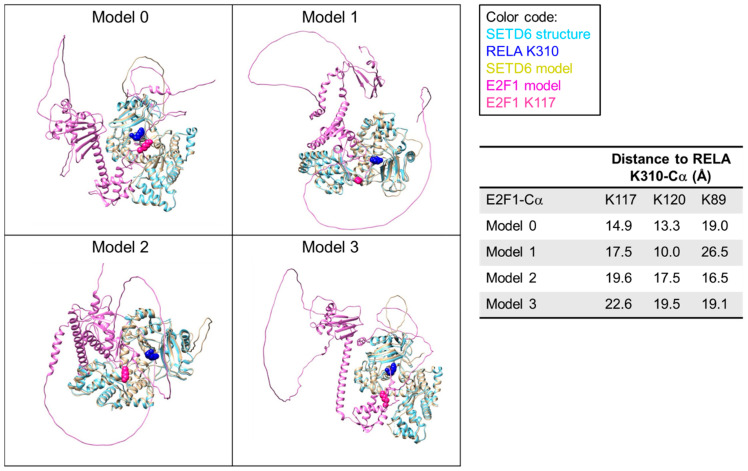
Compilation of AlphaFold 3 models of the SETD6–E2F1 complex. E2F1-K117 is highlighted in pink spheres. The models were superimposed with the SETD6-RELA structure (pdb 3qyx) [28] and K310 of RELA is shown as blue sphere to indicate the location of the SETD6 active site. Average pLDDT scores for different protein parts are provided in Supplementary Table S1.

A first level of SETD6 specificity can be explained by the pre-condition for active substrates to form an enzyme–substrate complex. Therefore, proteins which do not bind to SETD6 cannot be methylated, and the specificity of substrate binding by SETD6 serves as the first specificity filter. However, each of the bound substrate proteins typically is methylated by SETD6 at only one defined lysine residue while several other lysine residues are not methylated. One may speculate that the structure of the enzyme–substrate complex specifically places the target lysine into the active site of the PKMT while all other lysine residues are excluded. We have explored this hypothesis using the recently discovered E2F1 substrate of SETD6 as a test case [11] and modeled SETD6–E2F1 structures with AlphaFold 3 [25]. We inspected the four models of the SETD6–E2F1 complex with the highest scores and analyzed the approach of E2F1 to the active site of SETD6 and the proximity of different E2F1 lysine residues to the active site (Figure 1). All structures had SETD6 modeled with very good overlap to the known crystal structure [10] with RMSD values of the heteroatoms in the core region of the protein < 1Å and high pLDDT scores (~82, Supplementary Table S1). E2F1 was modeled with similar strong overlap to known structures, like the E2F1 (199–350) fragment [27], and high pLDDT scores for the structurally resolved part (~73, Supplementary Table S1). Moreover, all four AlphaFold 3 models showed that the E2F1 segment from R80 to T130 approaches the active site of SETD6, correctly identifying the E2F1 part that contains the target K117. However, the exact conformation and placement of the E2F1 residues 80–130 with respect to SETD6 differed in all four complexes. In agreement with this, the pLDDT scores of this part of E2F1 were low (~26, Supplementary Table S1).

As mentioned above, this part of E2F1 contains the K117 substrate residue and the distance of K117 to the active site was the smallest in the model with the best score. However, the 80–130 part of E2F1 also contains two more lysine residues (K89 and K120), neither of which is methylated by SETD6 [11]. Strikingly, in all structures at least one of these non-substrate lysine residues was closer to the active site of SETD6 than the true substrate K117. This flexibility in the enzyme–substrate interface is in agreement with the high K_M_ values typically seen for PKMT–peptide and protein interactions (e.g., in case of SETD6 the K_M_ for RELA peptide methylation is 55.2 µM and 16.6 µM for RELA protein methylation [29]) which are indicative of a relatively weak interaction. We conclude that there must occur some readout of the amino acid sequence surrounding potential target lysine residues that controls whether a lysine residue can finally dock into the active site in a catalytically competent conformation.

### 3.2. Peptide SPOT Array Methylation Experiments with SETD6

**Figure 2:**
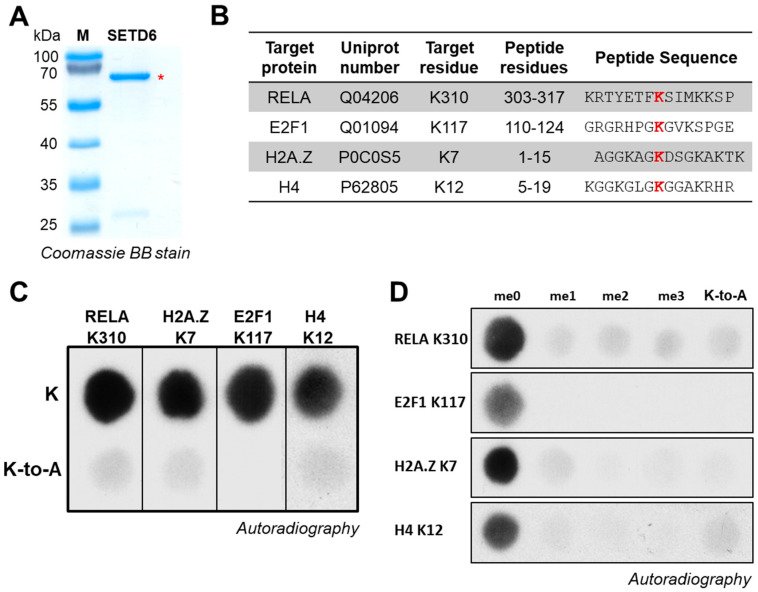
Purification of SETD6 and peptide methylation. (**A**) Coomassie-stained SDS-PAGE gel of purified SETD6. The band corresponding to SETD6 is indicated with a red asterisk. (**B**) Compilation of the RELA (aa 303–317), H2AZ (aa 1–15), E2F1 (aa 110–124), and H4 (aa 5–19) peptide sequences used in panels C and D. (**C**) Methylation of peptide SPOT arrays containing the sequences of the RELA, H2AZ, E2F1, and H4 peptide sequences (top row). Corresponding K-to-A mutant peptides are placed in the second row of the array. Film exposure times were 1 d for RELA, H2A.Z, and E2F1 and 7 h for the H4. (**D**) Methylation of peptide SPOT arrays containing variants of the peptides used in panel C with unmodified K, Kme1, Kme2, and Kme3 or K-to-A at the target position. Film exposure times were 1 d for RELA, 3 h for H2A.Z, 6 h for E2F1, and 7 h for H4.

To investigate the substrate recognition of SETD6 at the level of lysine residues interacting with its active site cleft, we analyzed the specificity of peptide methylation by purified SETD6 (Figure 2A). Peptide SPOT arrays presenting peptides covalently linked to a cellulose membrane provide powerful experimental tools to investigate the activity and specificity of PKMTs [23,24]. To study the activity of SETD6 in a diverse sequence context, we focused on the RELA K310 [10], E2F1 K117 [11], H2A.Z K7 [13], and H4 K12 [12] substrates (Figure 2B). First, pilot peptide arrays were prepared to validate the specific monomethylation of all substrate peptides at the target position (Figure 2C,D). Lack (or almost lack) of methylation of the K-to-A control peptides indicated that the additional lysine residues present in these sequences are not (or only very poorly) methylated by SETD6. Hence, the respective target lysine residues are the only lysine residues that are strongly methylated in the corresponding peptide.

The specificity of SETD6 was then studied by methylation of positional scanning SPOT arrays [23,24]. In these arrays, peptide libraries containing all peptide variants of an initial template sequence with single amino acid substitutions are synthesized on a cellulose membrane. The entire set of cellulose-bound peptides is then incubated with the PKMT in a suitable buffer containing AdoMet with a radioactively labeled methyl group and the methylation efficiency of all peptides is detected by autoradiography. This systematic mutational approach enables the comprehensive assessment of the methylation of peptides with all possible single-residue substitutions within the given sequence context. The resulting specificity profiles describe which amino acids are preferred and disfavored at each position of the substrate peptide. They provide detailed information on the molecular interaction of the PKMT with each amino acid in the substrate peptide.

### 3.3. Specificity of SETD6 for E2F1 K117

**Figure 3:**
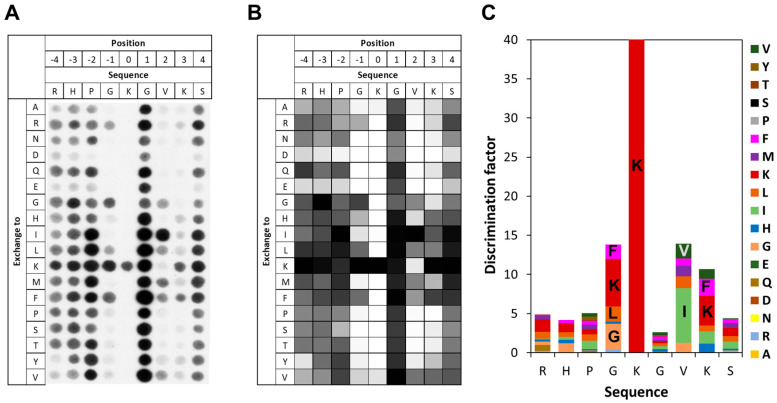
Specificity analysis of SETD6 based on the E2F1 peptide sequence. (**A**) Substrate sequence specificity scan conducted using E2F1 (110–124) as template sequence. The template sequence is represented on the horizontal axis, with target K117 at position 0. The vertical axis of the figure corresponds to the 18 amino acids, which were used for the generation of single amino acid mutants of the template sequence in the positional scanning array. The peptide array was methylated with SETD6 using radioactively labeled AdoMet, and the transfer of methyl groups was detected by autoradiography with a film exposure time of 2 days. (**B**) Quantitative analysis of two independent specificity scans. Autoradiography signals were quantified, normalized to the WT peptide at each position, and averaged across replicates. 91% of the peptides had an error < 25%. (**C**) Discrimination factor plot showing the relative preference of SETD6 for each amino acid at the positions −4 to +4 surrounding the target K. Although full-length 15-mer peptides were used in the array, analysis was restricted to this core recognition motif, where flanking sequence context plays a critical role in substrate recognition.

To investigate the specificity of SETD6 in the recognition of the E2F1 K117 residue, peptide SPOT arrays were synthesized using the sequence of E2F1 (residues 113–121) as template. The SPOT peptide arrays were incubated with SETD6 in the presence of radioactively labeled AdoMet and methylation was detected by autoradiography. As shown in Figure 3, SETD6 recognizes a substrate motif spanning from G116 (−1 site, when considering the target lysine K117 as position 0) to K120 (+3 site). SETD6 exhibited absolute specificity for K117 at the methylation site, indicating that this is the only lysine residue methylated in the peptide as already observed with the K-to-A mutant peptide of E2F1 in Figure 2C,D. Furthermore, a strong substrate preference was observed at the −1 site, mainly favoring K, followed by G, F, and L. At the +2 site, large aliphatic residues were preferred, with I most favored and followed by V. At the +3 site, the preferred residues are large and hydrophobic and/or positively charged with the highest preference for K, followed by F. Acidic residues (D and E) are disfavored at all −4 to +4 sites.

### 3.4. Specificity of SETD6 for H2A.Z K7

**Figure 4:**
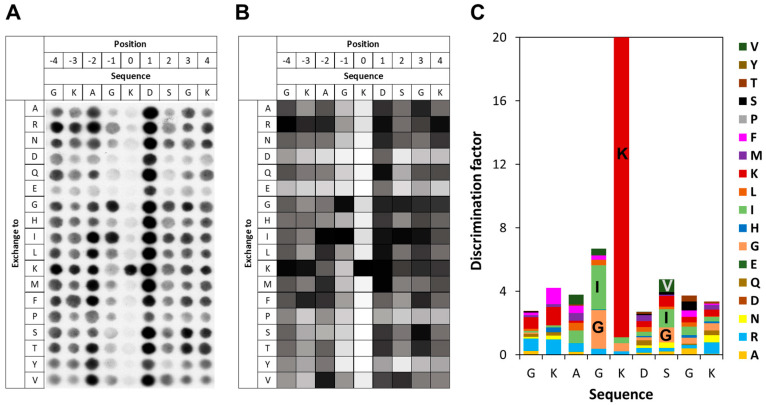
Specificity analysis of SETD6 based on the H2A.Z peptide sequence. (**A**) Substrate sequence specificity scan conducted using H2A.Z (1–15) as template sequence. Film exposure time was 1 day. For further details refer to the legend of Figure 3. (**B**) Quantitative analysis of two independent specificity scans. Autoradiography signals were quantified, normalized to the WT peptide at each position, and averaged across replicates. 95% of the peptides had an error < 25%. (**C**) Discrimination factor plot showing the relative preference of SETD6 for each amino acid at the positions −4 to +4 surrounding the target lysine.

To investigate the specificity of SETD6 in the context of the H2A.Z K7 target lysine, a mutational scanning SPOT peptide array was prepared using H2A.Z (residues 1–15) as a template sequence. The array was methylated and analyzed as described for the E2F1 arrays. As shown in Figure 4, in the H2A.Z context SETD6 recognizes a substrate motif mainly extending from the −1 to the +2 site with the target K7 defined as position 0. Notably, a pronounced substrate preference was observed at the −1 position, corresponding to G6 in the native H2A.Z sequence. Similar to the observations made in the E2F1 context at this position, SETD6 showed a strong preference for I and G in the H2A.Z sequence context. SETD6 exhibited strong preference for K7 at the methylation site, but some activity was also observed at peptides with mutated K7 indicating methylation of additional K residues in the peptide. This can be explained, because the peptide contains two more K residues, which even occur in preferred GK motifs. At the +2 site, large aliphatic residues like I, V, and also K are among the preferred ones, but the overall readout was weak and G was also among the preferred residues. Across all −3 to +4 sites, acidic residues (D and E) and proline were disfavored.

### 3.5. Specificity of SETD6 for RELA K310

**Figure 5:**
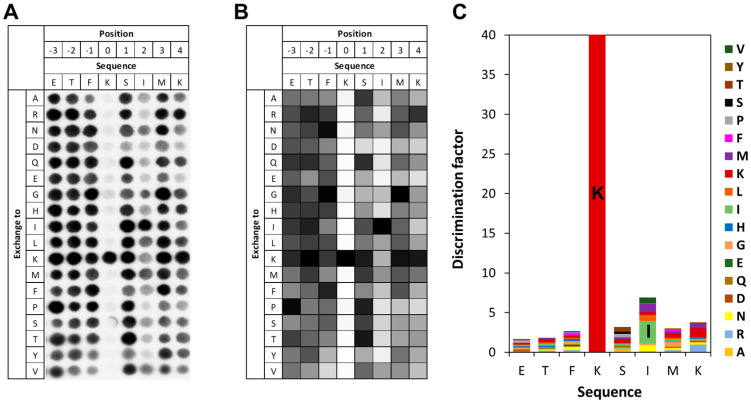
Specificity analysis of SETD6 based on the RELA peptide sequence. (**A**) Substrate sequence specificity scan conducted using RELA (303–317) as template sequence. Film exposure time was 1 day. For further details refer to the legend of Figure 3. (**B**) Quantitative analysis of two independent specificity scans. Autoradiography signals were quantified, normalized to the WT peptide at each position, and averaged across replicates. 96% of the peptides had an error < 25%. (**C**) Discrimination factor plot showing the relative preference of SETD6 for each amino acid at the positions −3 to +4 surrounding the target lysine.

To investigate the specificity of SETD6 in the context of the RELA K310 target lysine residue, a mutational scanning SPOT peptide array was prepared using RELA (residues 303–317) as template sequence. The array was methylated and analyzed as described for the E2F1 array. As shown in Figure 5, it revealed a very high specificity for K310 as a target residue, indicating that it is the only K residue methylated in the peptide. Moreover, there was sequence readout at the +2 site, where the natural I312 was preferred. No noticeable residue readout was detected at any other site, except a slight disfavor for D and E at the +1 to +4 sites.

### 3.6. Specificity of SETD6 for H4 K12

**Figure 6:**
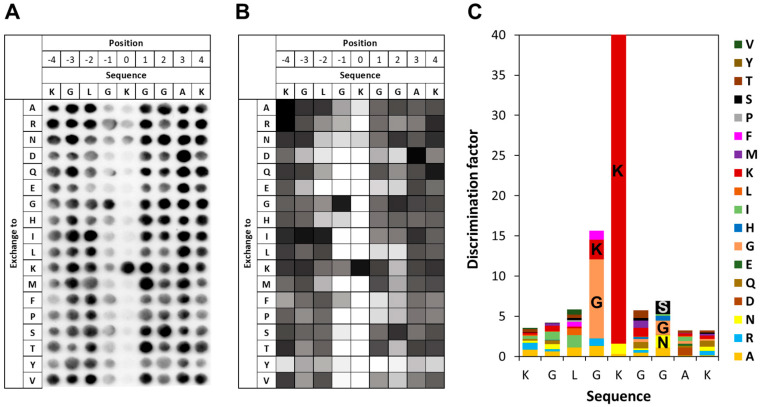
Specificity analysis of SETD6 based on the H4 peptide sequence. (**A**) Substrate sequence specificity scan conducted using H4 (5–19) as template sequence. Film exposure time was 1 day. For further details refer to the legend of Figure 3. (**B**) Quantitative analysis of two independent specificity scans. Autoradiography signals were quantified, normalized to the WT peptide at each position, and averaged across replicates. 90% of the peptides had an error < 25%. (**C**) Discrimination factor plot showing the relative preference of SETD6 for each amino acid at the positions −4 to +4 surrounding the target lysine.

To investigate the specificity of SETD6 in the context of the H4 K12 target lysine residue, a mutational scanning SPOT peptide array was prepared using H4 (residues 5–19) as a template sequence. The array was methylated and analyzed as described for the E2F1 array. As shown in Figure 6, in the H4 K12 context SETD6 recognizes a substrate motif mainly extending from the −1 to the +2 site. Similar to that observed with H2A.Z, SETD6 showed some activity at peptides with mutated K7, which can be explained by the presence of additional K residues in this peptide, one of them even in the preferred GK context. Moreover, a strong sequence readout was observed at the −1 position with a preference for G followed by K. Weak sequence recognition was detected at +2 with a preference for small hydrophilic residues (G, S, N, A). Other positions did not reveal noticeable sequence readout.

### 3.7. Role of L260 in SETD6 Substrate Recognition

**Figure 7:**
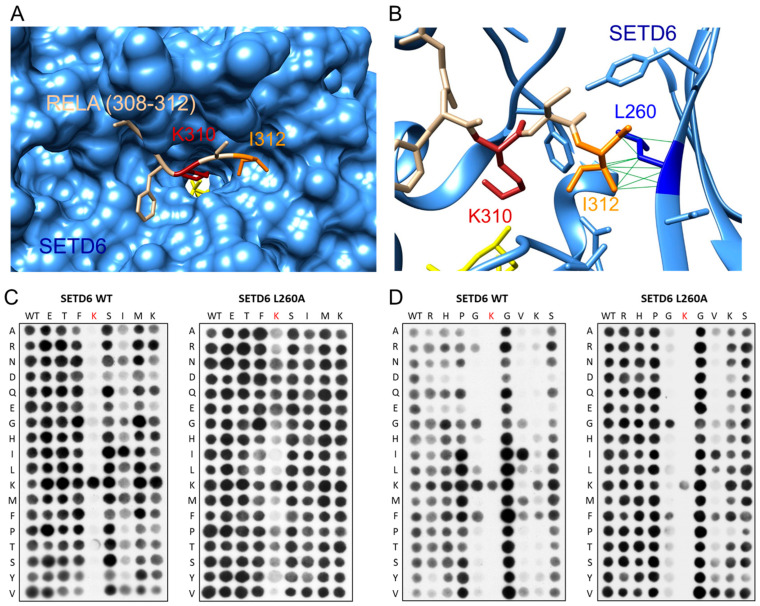
Investigation of the role of L260 in the substrate recognition of SETD6. (**A**) Structure of the SETD6-RELA (308–312) peptide complex (pdb 3RC0) [10]. SETD6 is shown as blue surface, the RELA peptide in tan. The target lysine K310 is shown in red, I312 in orange, and AdoMet in yellow. (**B**) Detail of the contact between RELA-I312 and SETD6-L260. SETD6 is shown in blue ribbon with residues contacting RELA-I312 shown as sticks. SETD6-L260 is shown in dark blue, all other colors are as in panel A. (**C**) Specificity analysis of WT SETD6 (taken from Figure 5A) and SETD6-L260A in the RELA sequence context. (**D**) Specificity analysis of WT SETD6 (taken from Figure 3A) and SETD6-L260A in the E2F1 sequence context. Both SETD6-L260 specificity arrays were conducted in two replicates with almost identical results.

A crystal structure is available of SETD6 with bound RELA peptide (308–312) (pdb 3RC0) [28]. It shows the peptide bound into a deep cleft of the SETD6 protein (Figure 7A). Our results reveal a specific recognition of E2F1-I312, which is located in a hydrophobic pocket with a particularly close interaction with SETD6-L260 (Figure 7B). We, therefore, have created and purified the SETD6-L260A mutant (Supplementary Figure S1) and analyzed its substrate specificity. As data with NSD1 and NSD2 indicated that enzyme mutations generating more space in the enzyme–peptide interface can lead to the catalysis of higher methylation states [30], we tested if L260A can also methylate substrates with Kme1 at the target site (Supplementary Figure S1C). However, this was not the case, suggesting that L260A like WT SETD6 can only introduce monomethylation.

Next, peptide recognition of L260A was tested using positional scanning SPOT arrays based on the RELA and E2F1 sequences, because these two substrates exhibited strong readout of large aliphatic residues at this site by SETD6 WT (Figure 3 and Figure 5). Surprisingly, the results of the L260A mutation were quite different in both sequence contexts. In the RELA context (Figure 7C), readout at the +2 position was completely lost in SETD6-L260A. As a consequence, the L260A mutant acted as an unspecific PKMT, as indicated by the observation that peptides with mutated K310 target lysine were still methylated, indicating that SETD6-L260A strongly methylates other lysine residues in the substrate peptide, something not observed with WT SETD6. In contrast, in the E2F1 context (Figure 7D), sequence readout of the L260A mutant was almost unchanged at the +2 site, but the preference for K at the +3 site was lost. Moreover, there were also subtle differences in the recognition at the −1 site, where the preference for K > G, F, L observed with WT SETD6 was changed to a preference for G > F in the case of SETD6-L260A. Additionally, the disfavor for E and D at −2 to −4 was also lost with the L260A mutant. However, there were no indications of an overall non-specific activity of SETD6-L260A in this sequence context.

## 4. Discussion

**Figure 8:**
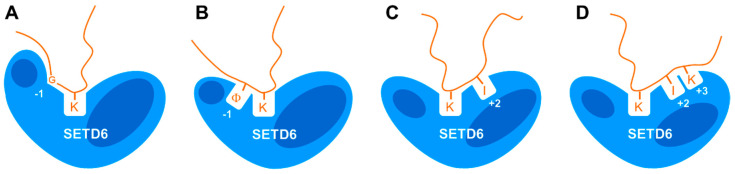
Compilation of the data. Illustration showing that SETD6 interacts with substrate peptides in different binding modes, leading to distinct substrate specificity profiles depending on the substrate sequence. (**A**) Recognition of G at the −1 site as observed with E2F1, H2A.Z, and H4. (**B**) Recognition of long aliphatic residues (symbolized by Φ) at the −1 site as observed with E2F1, H2A.Z, and H4. (**C**) Recognition of I at the +2 site as observed in E2F1 and RELA. (**D**) Recognition of I and K at the +2 and +3 sites as observed with E2F1.

In this work, we have systematically mapped the specificity of the amino acid recognition by SETD6 at different positions of the target peptide in the context of four substrates with different sequences. Our work has discovered three points of specific enzyme–substrate interactions, at the −1, +2, and +3 positions (Table 2 and Figure 8). At the −1 site, we observed a dual specificity, either for G or for K/I both sharing a long aliphatic part. Similarly, at the +2 position we observed a dual specificity either for I and V or for G. The main preference at +3 was for K. Of note, our findings indicate that these three contact points for recognition of substrate amino acids are not always used in the same way, but whether and how a particular contact is used or not depend on the substrate sequence as discussed below in more detail.

Readout at the −1 site was observed in the E2F1, H2A.Z, and H4 substrates but not with RELA. Interestingly, the natural amino acid residues found in all substrates with sequence readout at the −1 site at this place are glycine. RELA contains an F, which is the second-best residue observed with E2F1 at the −1 site, but interestingly the absence or presence of the F in the RELA peptide did not affect its methylation levels. Unexpectedly, at the −1 sites of the E2F1, H2A.Z, and H4 substrates, not only G was preferred but either K, I, or F and L also led to high methylation activity. Since the physicochemical properties of G are very different from K/I/F/L, this suggests that alternative catalytically competent conformations are possible in the N-terminal part of the substrate peptide. Either a specific conformation only accessible for G is adopted (indicated by a kink in the peptide at this position in Figure 8A) or the aliphatic parts of I and K interact with a hydrophobic binding pocket in SETD6 (Figure 8B). In addition, in the RELA substrate this part of the peptide is not recognized, either because the peptide is in a conformation not forming close interactions with SETD6 or SETD6 is in a conformation not exposing the hydrophobic binding pocket. A similar dual specificity for G or R at one sequence position has recently been observed for the PRDM9 PKMT [31]. In this case, we could show by biochemical experiments and molecular dynamics (MD) simulations that a binding tunnel in the enzyme can either accommodate the peptide backbone of the GG stretch or it interacts with the three methylene groups of the R, leading to a strong shift in the position and conformation of the substrate peptide depending on whether the GG or R is present. In addition, MD simulations and biochemical experiments have revealed that the SETD2 and NSD2 PKMTs methylate their natural H3K36 substrate and designed super-substrates with different transition state conformations [32,33,34].

Strikingly, the sequence readout of SETD6 at the +2 and +3 substrate sites was also strongly dependent on the substrate sequence context. Among all substrates, only RELA and E2F1 showed strong readout at the +2 site, in both cases favoring I although E2F1 naturally contains a V. H2A.Z showed weak dual specificity either preferring I/V or G, which is among the weakly preferred residues of H4-K12 at this site as well. A specific sequence interaction at the +3 site was only detected with the E2F1 substrate showing a preference for K. Hence, the C-terminal part of the substrate peptide either interacts with the +2 and +3 sites or only with the +2 site or with none of them and at the +2 site two interaction modes are possible. The variable relevance of the substrate amino acid binding interfaces at the −1, +2, and +3 sites suggests that the enzyme–substrate complex can adopt variable, substrate sequence-dependent transition state conformations which require a tight interaction of SETD6 with the substrate at some of these sites, while a flexible interface is provided at others, which does not support to sequence-specific substrate interactions. In the case of the RELA substrate, a tight interaction at the C-terminal part of the peptide (including the readout of I312 at the +2 site) may allow a flexible interface at the N-terminal region which does not support amino acid readout at the −1 site.

The opposite could be true for the H2A.Z and H4 substrates. Efficient methylation of these substrates depends on the tight interaction of the N-terminal parts with SETD6 including sequence recognition of the G at the −1 site, but it does not require strong interaction with the C-terminal part. Finally, our data show that efficient methylation of E2F1 requires a tight interaction of SETD6 with the N- and C-terminal parts of the substrate peptide. Future structural studies and/or MD simulations with WT and mutated SETD6 substrates may unravel the exact molecular details underlying these effects. Recently, the multispecific protein histidine methyltransferase CARNMT1 has been found to employ a similar mechanism of variable use of substrate recognition interfaces to allow specific interaction with different substrates [35], suggesting that it may also operate in other multispecific protein methyltransferases. Of note, this flexible recognition process involving conformational variability makes it difficult to predict novel substrates of protein methyltransferases from the consensus sequences of known targets.

In the SETD6-RELA structure, SETD6-L260 and RELA-I312 form a hydrophobic contact. We, therefore, studied the relevance of L260 in the recognition at the +2 site in the RELA and E2F1 context in more detail. Indeed, the L260A mutation converted SETD6 into a non-specific PKMT on the RELA peptide. This finding was expected, as I312 was the only residue accurately recognized in the RELA substrate, suggesting that loss of this contact should abrogate sequence recognition. However, unexpected results were obtained in the E2F1 context, because readout at the +2 position was almost not affected by the L260A mutation. Instead, the preference of SETD6 for K at the +3 site was lost, suggesting that in this amino acid sequence context L260 contacts the aliphatic chain of the K at position +3. Moreover, no unspecific activity of SETD6-L260A was observed on the E2F1 peptide which can be explained by the remaining contacts at the −1 and +2 positions which still ensure an accurate recognition of the target peptide and restrict the catalytic activity of the SETD6-L260A mutant to the original target lysine K117. Taken together, these results suggest that L260 has a different role in the substrate recognition of E2F1 than in RELA. They further illustrate the malleability of the SETD6–peptide interface that can adopt alternative catalytically active conformations with different contact patterns between the enzyme and substrate peptides in different amino acid sequence contexts.

## 5. Conclusions

The substrate specificity of PKMTs is driven by substrate protein binding and the specific interaction of the peptide region containing the target lysine with the active site cleft of the PKMT. Our data show that the peptide recognition by SETD6 is variable with the peptide sequence, where recognition interfaces may or may not be involved in the interaction with a certain substrate. Moreover, even within one sequence alternative catalytically competent conformations of the substrate peptide are possible. Our data explain the multispecificity of SETD6 by a malleability of the SETD6–peptide interface that can adopt alternative transition state conformations depending on the substrate amino acid sequences. We propose that the concept of substrate sequence-dependent transition state conformations is transferable to other multispecific protein methyltransferases.

## Figures and Tables

**Table 2:** Summary of the substrate peptide sequence recognition of SETD6 observed with the different substrates.

Substrate	Peptide Sequence (−3 to +3)	Preference at Position		
		−1	+2	+3
RELA K310	ETF**K**S**I**M		I	
E2F1 K117	HP**G****K**G**VK**	K > GFL	I > V	KF
H2A.Z K7	KA**G****K**DSG	GI	IVG	
H4 K12	GL**G****K**GGA	G > K	GSNA	

## Data Availability

All analyzed data are included in the published article and its supplementary information.

## Supplementary Materials

The following supporting information can be downloaded at: https://www.mdpi.com/article/doi/s1xxx, Supplementary Figure S1: Generation and purification of SETD6 and its L260A mutant; Supplementary Table S1: Compilation of averages of the atom specific pLDDT scores in the 4 best AlphaFold 3 models of the SETD6-E2F1 complex.
