# Supplementary material for: Mechanisms of Substrate Recognition by the Multispecific Protein Lysine Methyltransferase SETD6
Source: Life (Basel). 2025 Oct 10;15(10):1578. doi: 10.3390/life15101578 (PMC12565505; doi:10.3390/life15101578)
Supplement: Supplementary file 1 [file life-15-01578-s001.zip › life-3884876-supplementary.pdf]

Mechanisms of substrate recognition by the multispecific protein lysine methyltransferase SETD6

Supplementary Information

Supplementary Figure S1: Generation and purification of SETD6 and its L260A mutant. A) Validation of the SETD6 L260A mutant by Sanger sequencing. **B)** Coomassie-stained SDS-PAGE gel of the purified SETD6 WT and L260A proteins. The band corresponding to SETD6 is indicated with a red asterisk. **C)** Methylation of peptide spot arrays containing variants of the E2F1 substrate peptide with unmodified K, Kme1, Kme2, and Kme3 or K-to-A at the target position with the SETD6 L260A mutant.

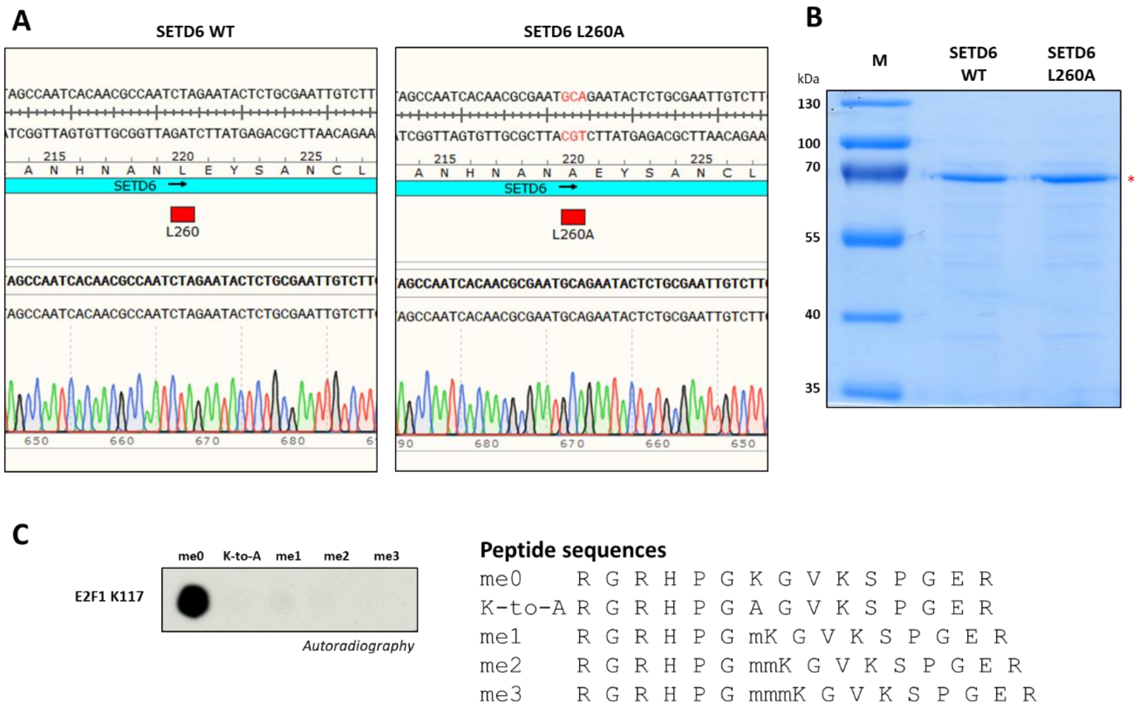

Supplementary Table S1: Compilation of averages of the atom specific pLDDT scores in the 4 best AlphaFold 3 models of the SETD6-E2F1 complex. Average pLDDT scores are provided for the part of E2F1 that docks into SETD6 (residues, 80-130) and contains the substrate lysine residue K117, as well as the part of E2F1 that is resolved in the pdb structure 2AZE [1] (residues 200-301). For SETD6, average pLDDT scores are provided for the part that is resolved in the pdb structure 3QXY [2] (residues 20-449).

	pLDDT score		
	E2F1 (80-130)	E2F1 (200-301)	SETD6 (20-449)
Model 0	26.5	73.7	81.3
Model 1	26.3	73.2	82.4
Model 2	26.6	73.6	81.9
Model 3	27.0	72.6	82.4

Regions with pLDDT scores above 70 are considered as high-quality predictions with generally accurate backbone structure, while regions with scores <50 are considered unreliable and likely disordered or flexible.

Supplementary references

1. Rubin, S.M.; Gall, A.L.; Zheng, N.; Pavletich, N.P. Structure of the Rb C-terminal domain bound to E2F1-DP1: a mechanism for phosphorylation-induced E2F release. *Cell* **2005**, *123*, 1093-1106, doi:10.1016/j.cell.2005.09.044.
2. Chang, Y.; Levy, D.; Horton, J.R.; Peng, J.; Zhang, X.; Gozani, O.; Cheng, X. Structural basis of SETD6-mediated regulation of the NF-kB network via methyl-lysine signaling. *Nucleic Acids Res* **2011**, *39*, 6380-6389, doi:10.1093/nar/gkr256.
